# *Lilium* spp. pollen in China (Liliaceae): Taxonomic and Phylogenetic Implications and Pollen Evolution Related to Environmental Conditions

**DOI:** 10.1371/journal.pone.0087841

**Published:** 2014-01-31

**Authors:** Yun-peng Du, Chi Wei, Zhong-xuan Wang, Shuang Li, Heng-bin He, Gui-xia Jia

**Affiliations:** College of Landscape Architecture, National Engineering Research Center for Flowers, Beijing Forestry University, Beijing, China; University of New South Wales, Australia

## Abstract

Recent molecular and karyologic studies have significantly modified delimitation of *Lilium*. However, despite the importance of pollen evolution in the genus comprehensive studies with electron microscopy and evaluation of pollen evolution are lacking. Therefore, we studied pollen morphology in a sample of 65 individuals from 37 taxa covering all the sections distributed in the world, using scanning electron microscopy. Our collection of 49 individuals from 21 taxa covering all five sections in China was also included in the database. We found pollen tetrads in *L. bakerianum.* Based on present and previous studies, our results suggest that pollen from *L. formosanum* should be classified as a new type, Formosanum. Combined with morphological and molecular evidence, pollen sculpture patterns appear to reflect phylogenetic relationships and are useful for species or subsection delimitation. Based on a comprehensive survey and correlation with potential functional implications, we propose the following hypothesis: evolution of an exine sculpture shows pollen type trends from Martagon → Callose → Concolor → Formosanum. The evolutionary trend regarding pollen sculpture and size could be related to selective pressure to adapt to environmental conditions. Pollen size and shape showed a significantly positive correlation with annual precipitation, and smaller pollen grains appear to adapt better in habitats with extreme conditions. Evolution trends in exine sculpture do not appear to be definitively correlated with pollen size and shape.

## Introduction

Approximately 110 to 115 *Lilium* species are distributed in the cold and temperate regions of the Northern Hemisphere [1], [2], particularly in East Asia, the Himalayas and Hengduan Mountains, North America and Europe. A total of 55 species occur in China [2]. De Jong [3] and Patterson and Givnish [4] consider southwest China and the Himalayas to be the center of origin of this genus.

Classification of this genus has been historically complicated. Several classifications for *Lilium* have been proposed based on morphological characters. Detailed studies have been performed for East Asia, European and North American *Lilium* species [5]–[7]. Based on 13 morphological characters and two germination types, Comber divided this genus into the following seven sections: *Martagon* Rchb., *Pseudolirium* Endl. which is limited to North America, *Liriotypus* Asch. and Graeb. which is distributed across Europe and the Caucasus, *Archelirion* Baker, *Sinomartagon* Comber, *Leucolirion* Wilson, and *Daurolirion* Comber, representing the most widely accepted taxonomical divisions [8]. Wang and Tang recognized sect. *Lophophorum* (Bur. et Franch.) Wang et Tang out of sect. *Sinomartagon* Comber and included campaniform-flowered species [9]. Liang [10] and Haw [11] modified sect. *Lophophorum* to accommodate the *Nomocharis*-like *Lilium* species in sect. *Sinomartagon* Comber. Chinese species were divided into five sections: *Martagon*, *Archelirion*, *Sinomartagon*, *Leucolirion* and *Lophophorum.*

Recently, molecular phylogenetic analyses and chromosome techniques have improved the understanding of several groups within the genus and modified the phylogenetic position of Comber’s classification, such as placement of sect. *Daurolirion* Comber in sect. *Sinomartagon*, *L. henryi* in subsect. *Leucolirion* 6b, modification of sect. *Lophophorum* and relationship confirmation in sect. *Liriotypus* [12]–[13], [16]–[21]. Preliminary research found that sect. *Sinomartagon,* which mainly occurred in China, was complicated and polyphyletic. As indicated by Patterson and Givnish [4], intercontinental dispersal details of the genus *Lilium* are not yet clear. The division of subsect. *Sinomartagon* 5c and sect. *Lophophorum* are controversial and will require further research. Nishikawa et al. [18], [19] suggested that *L. henryi* be classified into subsect. *Leucolirion* 6a and that it showed similar morphological features with *L. rosthornii*, thereby demonstrating that the phylogenetic position of *L. rosthornii* needs further study.

There are few relevant studies regarding pollen morphology which defines the taxonomic and reflect the evolution of the genus. According to the description within *Lilium* by Baranova [22] based on the number, shape and arrangement of columellae that form the muri, there are three morphological types of pollen: (1) Martagon (muri formed by rectangular columellae); (2) Callose (muri formed by rounded columellae); and (3) Concolor (muri formed by separated rounded and polygonal columellae). Previous studies found that most *Lilium* species have single pollen grains. However, pollen tetrads were found in *L. sempervivoideum* H. Lév. and *L. amoenum* E. H. Wilson ex Sealy, and the size and sculptural elements confirmed the taxa as two subspecies in *L. sempervivoideum* [23]. The pollen morphology of *L. lophophorum* (Bur. et Franch.) Franch., *L. henrici* Franch., *L. souliei* (Franch.) Sealy and *L. nanum* Klotz. et Garcke supported placement in *Lilium,* which differs from *Nomocharis* in apture and sculptural elements, and showed an evolutionary aperture trend from monocolpate to porate [24]. Muratović et al. [25] showed that two related European species, *L. bosniacum* and *L. carniolicum*, share similar pollen morphology. In addition, pollen morphology of some Chinese species under scanning electron microscopy (SEM), including 9 species described by Li and Qin [26], 10 species and 3 cultivars by Zhang et al. [27], and 12 species and 6 cultivars by Wu et al. [28], could provide taxonomic implications within *Lilium*:pollen has not only the commonness of genus, but also the specificity of single species. Interspecific pollen size and morphological characteristics has some difference, which has a certain reference value for the classification of *Lilium*. For example, there are differences in pollen size among *L. cernnum*, *L. lanciflium* and *L. pumilum*. Also there are significant differences in pollen ornamentation and morphology between *L. leucanthum* and its variety *L. leucanthum* var. *centifolium* from Qinling Mountains. Results obtained by Wang et al. [29] and Liu [30] indicate that pollen size parameters of *L. pumilum* and *L. concolor* from different provenances showed different degrees of variation. Determining whether this is a universal phenomenon in other species or if it is an important characteristic of species from different provenances requires a large amount of data. Therefore, a comprehensive study of *Lilium* pollen morphology in China, particularly a comparative study between populations or provenances using SEM, was needed. Environmental constraints act on patterns of differentiation, as all character states are the consequence of interactions between phylogenetic and environmental constraints [31]. Pollen could be considered a functional unit, with the exine structure as a compromise between four main functions [32]: protective, harmomegathic (the ability to absorb bending stresses, as occur during desiccation), reservoir (the role in producing an adhesive surface or as recognition substances) and clustering. Based on a comparative survey in the *Rhaponticum* group (Asteraceae, Centaureinae), Hidalgo et al. [33] hypothesized that occurrence in habitats with extreme conditions appears to be the main factor in the evolution from one form of pollen to another.

By combining previous and present results, our aim is to provide palynology information regarding the extent of pollen morphological diversity within and between species/sections, link this information with current phylogenetic data, and contribute to a comprehensive understanding of the systematics and evolution of *Lilium* by: (1) verifying pollen types in Chinese species; (2) discussing these findings within a phylogenetic framework; and (3) correlating pollen morphology and size with habitat.

## Materials and Methods

### Ethics Statement

For conservation reasons, we collected only small amounts of *Lilium* pollen. Our activities did not have any adverse effects of *Lilium* populations, and permission for collecting was obtained from National Engineering Research Center for Flowers of China (No.35 Tsinghua East Road Haidian District, Beijing, P.R. China).

Table 1 shows the origin of all species studied which were collected fresh. Our observations for 21 taxa represented by 49 samples were also included in the database. Division into the sections was based on studies by Comber [8], Gao et al. [13], Nishikawa et al. [18], [19] and Du et al. [16]) as follows: two species and one variety of sect. *Martagon*, nine species and three varieties of sect. *Sinomartagon* including *L. dauricum* Ker-Gawl (sect. *Daurolirion* Comber), eight species and one variety of sect. *Leucolirion* including *L. brownii* F. E. Brown ex Miellez (sect. *Archelirion* Comber), *L. henryi* Baker and *L. rosthornii* Diels (sect. *Sinomartagon* Comber), one species of sect. *Archelirion*, two species of sect. *Liriotypus*, three species of sect. *Pseudolirium*, and six species and one variety of sect. *Lophophorum* (Table 1).

**Table 1:** Origin of the material and numerical results of the study.

Samples	Voucher	*P*(µm) *	*E*(µm)^#^	P/E	Lumina(µm)	Muri width(µm)	Pollentype**
*L.lancifolium* Thunb.	Du 08001, Fushun, Liaoning, China	108.20±7.57	41.55±4.39	2.62±0.21	4.24±1.21	1.22±0.20	Martagon
*L.lancifolium* Thunb.	Wang 11001, Shengnongjia, Hubei, China	113.20±8.69	40.14±2.67	2.83±0.22	6.76±2.04	1.50±0.19	Martagon
*L.lancifolium* Thunb.	Du 09001, Foping, Shaanxi, China	110.97±7.80	38.95±2.82	2.86±0.22	6.84±1.98	1.49±0.17	Martagon
*L.lancifolium* Thunb.	Du 11001, Chongqing, China	111.22±11.17	40.76±3.68	2.74±0.30	6.75±1.94	1.43±0.21	Martagon
*L.lancifolium* Thunb.	Du 09002, Hanzhong, Shaanxi, China	111.25±7.94	41.23±4.12	2.72±0.26	4.89±1.24	1.37±0.25	Martagon
*L.lancifolium* Thunb.	Du 11002, Yichang, Hubei, China	109.59±7.84	42.08±4.67	2.62±0.20	6.58±2.07	1.37±0.18	Martagon
*L.lancifolium* Thunb.	Du 11003, Zigui, Hubei, China	109.91±14.7	42.88±5.24	2.57±0.29	7.95±2.50	1.35±0.20	Martagon
*L.lancifolium* Thunb.	Du 09003, Taibai, Shaanxi, China	111.91±9.26	42.65±4.05	2.64±0.23	6.45±1.93	1.49±0.25	Martagon
*L.lancifolium* Thunb.	Wang 11002, Yichang, Hubei, China	113.72±8.91	39.62±3.35	2.88±0.28	6.64±2.26	1.35±0.23	Martagon
*L.lancifolium* Thunb.	Du 11004, Yichang, Hubei China	112.03±6.39	41.74±3.2	2.70±0.24	8.15±2.78	1.48±0.21	Martagon
*L.distichum* Nakai etKamibayashi	Jia 07001, Changbai Mountains, Jilin, China	88.34±4.52	32.90±2.66	2.70±0.23	8.29±1.97	1.46±0.17	Martagon
*L.distichum* Nakai etKamibayashi	Du 11005, Fushun, Liaoning, China	94.78±5.78	36.23±2.64	2.62±0.17	6.86±3.63	1.49±0.20	Martagon
*L.tsingtauense* Gilg	Du 11006, Tsingtao, Shandong, China	104.70±6.16	44.52±2.23	2.36±0.17	10.06±2.27	1.64±0.35	Martagon
*L.dauricum* Ker-Gawl.	Du 11007, Fushun, Liaoning, China	99.21±4.72	37.85±4.29	2.64±0.24	6.57±2.64	1.54±0.20	Martagon
*L.davidii* Duchartre ex Elwes	Wang 11002, Baoxing, Sichuan, China	95.50±4.35	33.36±2.44	2.88±0.23	6.18±2.15	1.28±0.16	Martagon
*L.davidii* Duchartre ex Elwes	Du 11008, Zigui, Hubei, China	94.22±5.11	34.48±2.82	2.75±0.21	5.38±1.87	1.17±0.14	Martagon
*L.davidii* Duchartre ex Elwes	Wang 11003, Chongqing, China	85.56±6.34	32.45±3.95	2.66±0.29	5.67±1.88	1.15±0.16	Martagon
*L.davidii* Duchartre ex Elwes	Du 11009. Lijiang, Yunnan, China	97.07±5.95	33.92±2.15	2.87±0.17	6.78±1.96	1.45±0.96	Martagon
*L.davidii* var. *willmottiaee*(E. H. Wilson) Raffill	Jia 07002, Lanzhou, Gansu, China	95.64±7.13	32.75±3.00	2.93±0.22	5.27±0.56	1.23±0.21	Martagon
*L.davidii* var.*willmottiaee*(E. H. Wilson) Raffill	Wang 11004, Heqing, Yunnan, China	88.14±3.82	34.43±2.28	2.57±0.26	5.79±1.51	1.20±0.14	Martagon
*L.duchartrei* Franch.	Wang 11005, Baoxing, Sichuan, China	85.07±10.81	33.31±3.70	2.56±0.23	8.31±2.79	1.30±0.15	Martagon
*L. leichtlinii* Hook.f. var.*maximowiczii* (Regel) Baker	Du 12001, Beijing Botanical Garden, Beijing, China	76.86±5.36	31.45±3.03	2.45±0.18	5.78±2.55	1.21±0.14	Martagon
*L. leichtlinii* Hook.f. var.*maximowiczii* (Regel) Baker	Du 11010, Fushun, Liaoning, China	88.75±5.26	34.63±2.54	2.58±0.23	7.39±2.31	1.29±0.17	Martagon
*L. concolor* Salisb. var.*pulchellum* (Fisch.) Regel	Du 11011, Fushun, Liaoning, China	71.03±4.66	30.56±2.42	2.34±0.21	5.71±1.53	1.21±0.36	Concolor
*L. concolor* Salisb. var.*pulchellum* (Fisch.) Regel	Du 11012, Fushun, Liaoning, China	70.51±5.13	30.59±2.44	2.31±0.11	3.50±1.79	1.50±0.20	Concolor
*L.pumilum* DC.	Jia 09001, Tongliao, Inner Mongolia, China	87.39±7.00	32.66±1.82	2.67±0.12	4.96±1.90	1.35±0.22	Martagon
*L.taliense* Franch.	Jia 09002, Chongqing, China	79.54±8.11	29.91±2.39	2.59±0.26	6.12±2.03	1.15±0.18	Martagon
*L.taliense* Franch.	Du 12002, Shangri-la,Yunna, China	69.85±3.51	29.49±3.42	2.40±0.27	3.57±1.1	1.07±0.09	Martagon
*L.wardii* Stapf ex Stearn	Du 11013, Bomi, Tibet	87.77±5.97	35.26±1.97	2.49±0.18	7.48±2.20	1.46±0.15	Martagon
*L.bakerianum* Coll. etHemsl	Du 12003, Lijiang, Yunnan, China	78.40±4.88	79.71±15.81	1.03±0.19	5.84±3.42	1.46±0.35	Concolor
*L.brownii* F. E. Brown exMiellez	Wang 11006, Shennongjia, Hubei, China	120.13±3.65	53.16±6.50	2.29±0.29	12.29±4.49	1.69±0.18	Martagon
*L.brownii* F. E. Brown exMiellez	Du 09004, Foping, Shaanxi, China	103.18±9.16	46.11±4.43	2.25±0.20	7.95±2.74	1.68±0.24	Martagon
*L.brownii* F. E. Brown exMiellez	Du 11014, Yichang, Hubei, China	109.77±4.24	50.91±5.39	2.18±0.23	10.44±4.37	1.86±0.28	Martagon
*L.henryi* Bake	Du 11015, Zigui, Hubei, China	92.67±4.65	35.98±2.70	2.59±0.18	4.36±2.40	1.11±0.15	Martagon
*L.henryi* Bake	Du 12004, Beijing botanical garden, Beijing, China	96.47±5.35	34.86±2.62	2.78±0.20	6.46±2.03	1.37±0.31	Martagon
*L.rosthornii* Diels	Jia 10001, Chongqing, China	98.01±5.87	35.20±2.38	2.79±0.17	5.08±1.60	1.06±0.11	Martagon
*L.rosthornii* Diels	Du 12005, Yuanling, Hunan, China	92.31±6.88	32.88±2.28	2.81±0.18	6.40±2.20	1.20±0.26	Martagon
*L. regale* Wilson	Du 11015, Maoxian, Sichuan, China	100.68±6.00	41.09±3.76	2.47±0.24	9.32±2.85	1.57±0.29	Callose
*L. regale* Wilson	Wang 11007, Lixian, Sichuan, China	93.55±4.40	42.62±2.97	2.20±0.15	9.94±3.48	2.22±0.46	Callose
*L. regale* Wilson	Du 11016, Wenchuan, Sichuan, China	95.78±5.15	39.95±2.88	2.41±0.21	10.26±3.55	1.52±0.47	Callose
*L.regale* Wilson	Du 12006, Beijing botanical garden, Beijing, China	99.33±5.16	40.16±3.92	2.49±0.22	8.93±2.91	1.79±0.25	Callose
*L.sargentiae* Wilson	Jia 11001, Chongqing, China	91.33±4.65	33.03±2.50	2.78±0.25	6.29±1.83	1.08±0.21	Callose
*L.sargentiae* Wilson	Du 11017, Baoxing, Sichuan, China	95.11±7.01	35.96±2.25	2.65±0.18	6.49±1.97	1.16±0.16	Callose
*L.sargentiae* Wilson	Du 11018, Baoxing, Sichuan, China	89.83±5.75	33.60±2.36	2.68±0.15	6.50±1.95	1.35±0.14	Callose
*L.sargentiae* Wilson	Wang 11008, Chongqing, China	92.10±4.90	35.67±2.92	2.60±0.26	6.52±1.95	1.66±0.23	Callose
*L.sargentiae* Wilson	Wang 11009, Chongqing, China	89.08±6.62	33.28±2.33	2.68±0.20	7.34±2.00	1.33±0.19	Callose
*L.leucanthum* (Baker)Baker	Du 11019, Zigui, Hubei, China	98.82±9.59	36.55±3.72	2.71±9.16	6.80±2.05	1.46±0.18	Callose
*L.sulphureum* Bakerapud Hook. f.	Du 12007, Beijing botanical garden, Beijing, China	87.56±4.58	38.69±3.58	2.28±0.23	6.40±2.01	1.50±0.30	Callose
*L.formosanum* Wallace	Du 12008, Beijing botanical garden, Beijing, China	122.30±7.64	49.89±4.22	2.46±0.20	12.63±3.71	1.22±0.39	Formosanum(this study)
*L.cernuum* Kom.	Zhang et al.(2006)	108.9	44. 9	2.43	4.99	2.1	Martagon
*L. amabile* Palib.	Zhang et al.(2006)	106.2	41. 8	2.54	7.55	1.93	Martagon
*L.leucanthum* var. *centifolium*(Stapf ex Elwes) Stearn	Zhang et al.(2010)	74.3	35.1	2.12	3.3	2.4	Martagon
*L. henricii* Franch.	Liang and Zhang (1985)	70.5	47.0				Concolor
*L.lophophorum*(Bur.et Franch.)Franch	Liang and Zhang (1985)	70.5	65.8				Martagon
*L.nanum* Klotz. Et Garche	Liang and Zhang (1985)	84.6	61.1				Martagon
*L.nanum* Klotz. Et Garche var. *brevistylum* S.L. Liang	Liang and Zhang (1985)	58.8	49.4				Martagon
*L.sempervivoideum* Levl.	Liang and Zhang (1984)	103.4	117.5				Martagon
*L. amoenum* Wilson ex Sealy	Liang and Zhang (1984)	112.8	122.2				Martagon
*L. speciosum* Thunb. var.*gloriosoides* Baker	Wu et al. (2007)	59.5	21.4	2.78	4.68	1.03	Martagon
*L. martagon* L.	Halbritter (1993)						Martagon
*L. bosniacum* (Beck)Beck ex Fritsch	Muratović et al. (2010)						Martagon
*L. carniolicum* Bernh. exW.D.J. Koch	Muratović et al. (2010)						Martagon
*L.canadense*	Kosenko (1999)	74.8–76.8	51.8–55.8				Martagon
*L.columbianum*	Kosenko (1999)	71.0–76.8	44.1–46.0				Martagon
*L.kesselringianum*	Kosenko (1999)	101.8–107.5	53.7–59.5				Martagon

Note: * Polar axis; # Equatorial axis; ** Following the nomenclature of Baranova (1985).

To compare pollen features at the genus level, a database with results of the present study and those of Halbritter [34], Kosenko [35], Liang et al. [23], [24], Muratović et al. [25], Wu et al. [28], Zhang et al. [27] and Zhang et al. [36] was established (Table 1). Because the results do not permit a calculation of all the parameters cited above, our observations from 49 samples representing 21 taxa was used only for statistical analyses.

Morphological observations were conducted using SEM. Pollen was directly attached to double-sided adhesive tape and examined under the microscope to locate the pollen. The sample was then taped to the object stage. Following gold spray coating, observation and image acquisition were conducted using a Hitachi S-3400 scanning electron microscope following the method described by Avetissian [37]. All microscopy procedures were performed at the Biotechnology Centre, Beijing Forestry University, China.

Biometric measurements were made using Image-Pro Plus 6.0 (Media Cybernetics, USA). For each sample, 10 (5 for *L. brownii* from Shennongjia, Hubei Province) fully developed pollen grains were measured. Parameters considered were the polar axis (*P*), equatorial diameter (*E*), *P*/*E* ratio, lumina diameter and muri width (Table 1). The description of pollen morphology was based on the shape and sculpturing classifications of Baranova [22]. Arithmetic mean and standard deviations are shown for each parameter.

To investigate pollen evolution, pollen features from the database were superimposed onto a phylogenetic framework constructed by internal transcribed spacer (ITS) sequences. We selected 23 taxa, including *Cardiocrinum gigantum* as an outgroup based on preliminary study [16]. The other 16 taxa, including an outgroup of *Northolirion bulbiliferum,* were cited [13], [18]–[20]. All of the sequence data from the ITS regions were double-checked visually, edited in BioEdit 5.0.6 [38], and aligned in Clustal X 1.83 [39] using default settings. Phylogenetic analyses were performed using maximum parsimony (MP) and maximum likelihood (ML). The MP tree was constructed with PAUP* 4.0b10 [40]. ML phylogenetic analysis was performed using RAxML v. 7.0.4 with unique model parameters for ITS sequences [41]. A general time reversible model (GTR) was applied with discrete gamma distribution. Bootstrap pseudo replicates were performed 1000 times using the fast bootstrapping option and the best scoring ML tree. Phylogenetic trees were visualized using Treeview [42]. The best scoring tree was visualized with FigTree v1.3.1 (http://tree.bio.ed.ac.uk/).

SPSS 18.0 was used for statistical analyses. One-way analysis of variance (ANOVA) was used to evaluate whether or not differences in pollen characteristics from different provenances were significant. In those cases in which ANOVA revealed significant differences, a least significant difference (LSD) test was performed (Table 2). Correlation analysis was used to evaluate the relationship between pollen parameters and habitat (Our collection of 21 taxa, including 49 samples) (Table 3 and 4).

**Table 2:** Variance analysis of pollen morphology traits among species from different provenances.

Traits	Species										
	*L.lancifolium*	*L.regale*	*L.davidii*	*L.sargentiae*	*L.brownii*	*L.davidii* var. *willmottiaee*	*L.distichum*	*L.henryi*	*L. concolor* var. *pulchellum*	*L.rosthornii*	*L. leichtlinii* var. *maximowiczii*
*P*	0.551	9.278**	14.763**	3.613**	10.40**	6.705*	20.757**	6.786*	0.086	7.964**	31.007**
*E*	2.426*	2.830*	1.526	6.811**	5.539**	2.068	23.439**	2.061	0.001	10.597**	6.793*
P/E	3.764**	9.996**	3.347*	1.8	0.61	14.288**	2.165	12.115**	0.186	0.164	2.742
Lumina	10.289**	0.837	5.638**	1.502	14.393**	2.994	5.163*	41.913**	66.946**	16.479**	17.303**
Muri	3.62**	51.346**	36.556**	65.017**	14.983	0.169	0.426	0.017	4.616*	21.425**	6.474*

Note: * represents the significant difference (p<0.05), and ** represents the extremely significant difference (p<0.01).

**Table 3:** Geographical and climate factors of provenances studied.

Samples	Voucher	Latitude (°)	Longitude (°)	Altitude (m)	Annual average temperature (°C)	Annual average sunshine (h)	Yearly precipitation (mm)
*L. lancifolium* Thunb.	Du 08001, Fushun, Liaoning, China	42.09	124.43	450	7	780	2300
*L.lancifolium* Thunb.	Wang 11001, Shengnongjia, Hubei, China	39.59	116.12	1200	12	1600	1858
*L. lancifolium* Thunb.	Du 09001, Foping, Shaanxi, China						
*L. lancifolium* Thunb.	Du 11001, Chongqing, China						
*L. lancifolium* Thunb.	Du 09002, Hanzhong, Shaanxi, China						
*L. lancifolium* Thunb.	Du 11002, Yichang, Hubei, China	31.00	110.58	721	13.6	1500	1669
*L. lancifolium* Thunb.	Du 11003, Zigui, Hubei, China	31.05	110.54	1510	13.6	1500	1669
*L. lancifolium* Thunb.	Du 09003, Taibai, Shaanxi, China						
*L. lancifolium* Thunb.	Wang 11002, Yichang, Hubei, China	31.01	110.57	1018	13.6	1500	1669
*L. lancifolium* Thunb.	Du 11004, Yichang, Hubei China	31.02	110.57	1110	13.6	1500	1669
*L. distichum* Nakai et Kamibayashi	Jia 07001, Changbai Mountains, Jilin, China	42.07	124.45	450	7	780	2300
*L. distichum* Nakai et Kamibayashi	Du 11005, Fushun, Liaoning, China	41.47	124.44	475	7	780	2300
*L. tsingtauense* Gilg	Du 11006, Tsingtao, Shandong, China	36.09	120.37	426	12.1	775.6	2503
*L. dauricum* Ker-Gawl.	Du 11007, Fushun, Liaoning, China	42.06	124.48	365	7	780	2300
*L. davidii* Duchartre ex Elwes	Wang 11002, Baoxing, Sichuan, China						
*L. davidii* Duchartre ex Elwes	Du 11008, Zigui, Hubei, China	31.05	110.53	1557	13.6	1500	1669
*L. davidii* Duchartre ex Elwes	Wang 11003, Chongqing, China	29.03	107.12	883	17	1395	1079
*L. davidii* Duchartre ex Elwes	Du 11009. Lijiang, Yunnan, China	41.34	122.32	2533	16.5	1000	2400
*L. davidii* var. *willmottiaee* (E. H. Wilson Raffill	Jia 07002, Lanzhou, Gansu, China						
*L. davidii* var. *willmottiaee* (E. H. Wilson) Raffill	Wang 11004, Heqing, Yunnan, China	26.19	100.09	2408	15.5	1050	2253
*L. duchartrei* Franch.	Wang 11005, Baoxing, Sichuan, China	30.50	102.43	2859	14.1	994	789
*L. leichtlinii* Hook.f. var. *maximowiczii* (Regel) Baker	Du 12001, Beijing Botanical Garden, Beijing, China	41.46	124.42	324	7	780	2300
*L. leichtlinii* Hook.f. var. *maximowiczii* (Regel) Baker	Du 11010, Fushun, Liaoning, China	41.46	124.43	388	7	780	2300
*L. concolor* Salisb. var. *pulchellum* (Fisch.) Regel	Du 11011, Fushun, Liaoning, China	42.06	124.47	330	7	780	2300
*L. concolor* Salisb. var. *pulchellum* (Fisch.) Regel	Du 11012, Fushun, Liaoning, China	41.35	124.09	324	7	780	2300
*L. pumilum* DC.	Jia 09001, Tongliao, Inner Mongolia, China	33.24	108.76	813	10	938	1726
*L. taliense* Franch.	Jia 09002, Chongqing, China	29.03	107.12	1265	17	1395	1079
*L. taliense* Franch.	Du 12002, Shangri-la,Yunna, China	27.52	99.49	3191	11	650	2000
*L. wardii* Stapf ex Stearn	Du 11013, Bomi, Tibet	30.06	95.04	2053	8.5	977	1563
*L. bakerianum* Coll. et Hemsl	Du 12003, Lijiang, Yunnan, China	26.28	100.43	1848	13.6	600	2403
*L.brownii* F. E. Brown ex Miellez	Wang 11006, Shennongjia, Hubei, China	39.59	116.12	1200	12	1600	1858
*L.brownii* F. E. Brown ex Miellez	Du 09004, Foping, Shaanxi, China	33.24	108.76	813	10	938	1726
*L.brownii* F. E. Brown ex Miellez	Du 11014, Yichang, Hubei, China	31.01	110.57	1004	13.6	600	2403
*L. henryi* Bake	Du 11015, Zigui, Hubei, China						
*L. henryi* Bake	Du 12004, Beijing botanical garden, Beijing, China	28.49	110.23	875	13.6	600	2403
*L. rosthornii* Diels	Jia 10001, Chongqing, China						
*L. rosthornii* Diels	Du 12005, Yuanling, Hunan, China	27.54	99.57	396	16.6	1437	1200
*L. regale* Wilson	Du 11015, Maoxian, Sichuan, China	31.42	103.51	1504	11.2	491	
*L. regale* Wilson	Wang 11007, Lixian, Sichuan, China	31.26	103.09	1910	9	800	
*L. regale* Wilson	Du 11016, Wenchuan, Sichuan, China	31.27	103.33	1409	14	920	
*L. regale* Wilson	Du 12006, Beijing botanical garden, Beijing, China						
*L. sargentiae* Wilson	Jia 11001, Chongqing, China	29.03	107.12	918	17	1396	1079
*L. sargentiae* Wilson	Du 11017, Baoxing, Sichuan, China	30.36	102.84	1015	14.1	994	789
*L.sargentiae* Wilson	Du 11018, Baoxing, Sichuan, China						
*L.sargentiae* Wilson	Wang 11008, Chongqing, China	29.04	107.16	787	17	1396	1079
*L. sargentiae* Wilson	Wang 11009, Chongqing, China						
*L. leucanthum* (Baker) Baker	Du 11019, Zigui, Hubei, China	31.05	110.54	1525	13.6	1500	1669
*L. sulphureum* Baker apud Hook. f.	Du 12007, Beijing botanical garden, Beijing, China						
*L. formosanum* Wallace	Du 12008, Beijing botanical garden, Beijing, China						

Note: Climate parameters were obtained from the local meteorological bureau.

**Table 4:** Correlation coefficient between the main characteristic of pollen morphology and the geographical and climate factors of provenances.

Traits	Latitude (°)	Longitude (°)	Altitude (m)	Annual average temperature (°C)	Yearly precipitation (mm)	Annual average sunshine (h)
*P*	0.046	0.082	−0.013	0.172	0.413*	−0.013
*E*	−0.105	−0.103	0.041	0.014	−0.129	0.249
P/E	0.133	0.178	−0.137	0.160	0.442**	−0.251
Lumina	0.010	−0.073	0.039	0.073	0.024	−0.091
Muri	0.126	0.048	−0.035	−0.199	−0.189	0.134

Note: * represents the significant difference (p<0.05), and ** represents the extremely significant difference (p<0.01).

## Results

Numerical results of the palynological study are summarized in Table 1. According to the *Lilium* classification by Comber [8], Nishikawa et al. [18], [19] and Du et al. (2013) [16], palynological results for each section are as follows:

### Martagon

**Figure 1:**
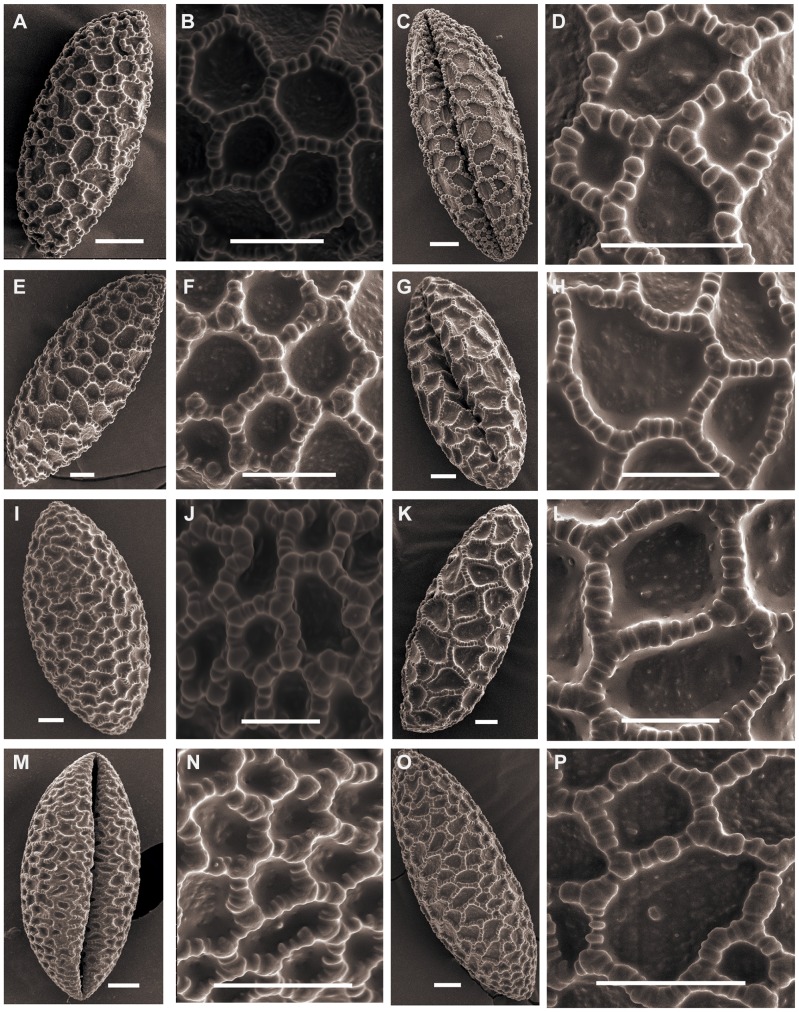
SEM photographs of pollen grains of *Lilium*. Figs A, B, *L. distichum* (Jia 07001). Figs C, D, *L. distichum* (Du 11005). Figs E, F, *L. tsingtauense*. Figs G, H, *L. brownii* (Wang 11006). Figs I, J, *L. brownii* (Du 09004). Figs K, L, *L. brownii* (Du 11014). Figs M, N, *L. lancifolium* (Du 08001). Figs O, P, *L. lancifolium* (Wang 11001). Scale bars: 10 µm.

Representatives of *Martagon* (Figure 1A–F) have pollen size ranging from *L. distichum* Nakai et Kamibayashi (91.56×34.57 µm, P/E of 2.66) to *L. tsingtauense* Gilg (104.70×44.52 µm, P/E of 2.36). The shape can be long-ellipsoidal. *L. tsingtauense* and *L. distichum* pollen show similar exine sculptures with a slight difference in that the intersection of the muri presents a protuberant and enlarged columellae in *L. tsingtauense* pollen (Figure 1E–F). *L. tsingtauense* and *L. distichum* correspond to Martagon type pollen, which is in agreement with Baranova [22].

### Sinomartagon

**Figure 2:**
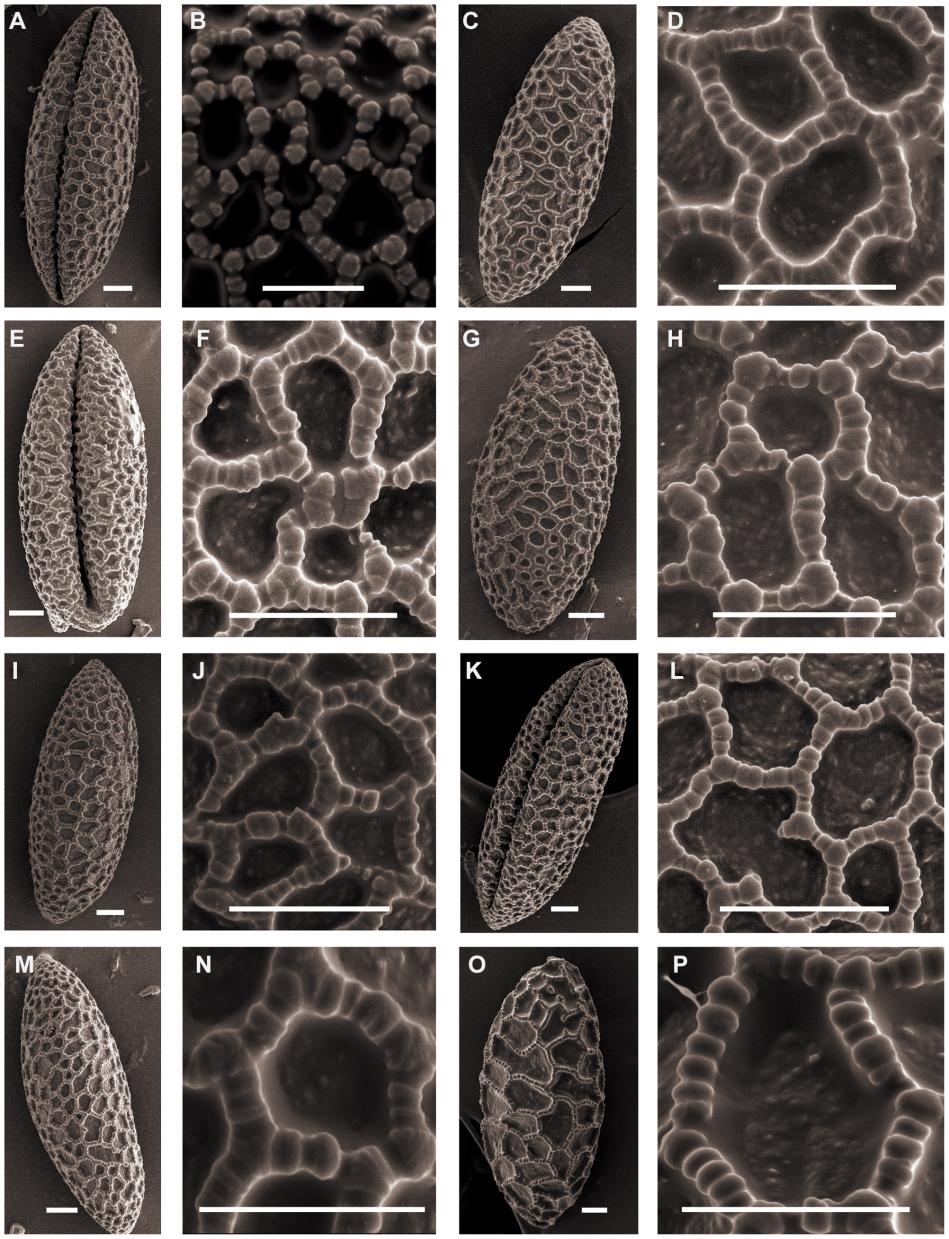
SEM photographs of pollen grains of *Lilium*. Figs A, B, *L. lancifolium* (Du 09001). Figs C, D, *L. lancifolium*(Du 11001). Figs E, F, *L. lancifolium* (Du 09002). Figs G, H, *L. lancifolium* (Du 11002). Figs I, J, *L. lancifolium* (Du 11003). Figs K, L, *L. lancifolium* (Du 09003). Figs M, N, *L. lancifolium* (Wang 11002). Figs O, P, *L. lancifolium* (Du 11004). Scale bars: 10 µm.

**Figure 3:**
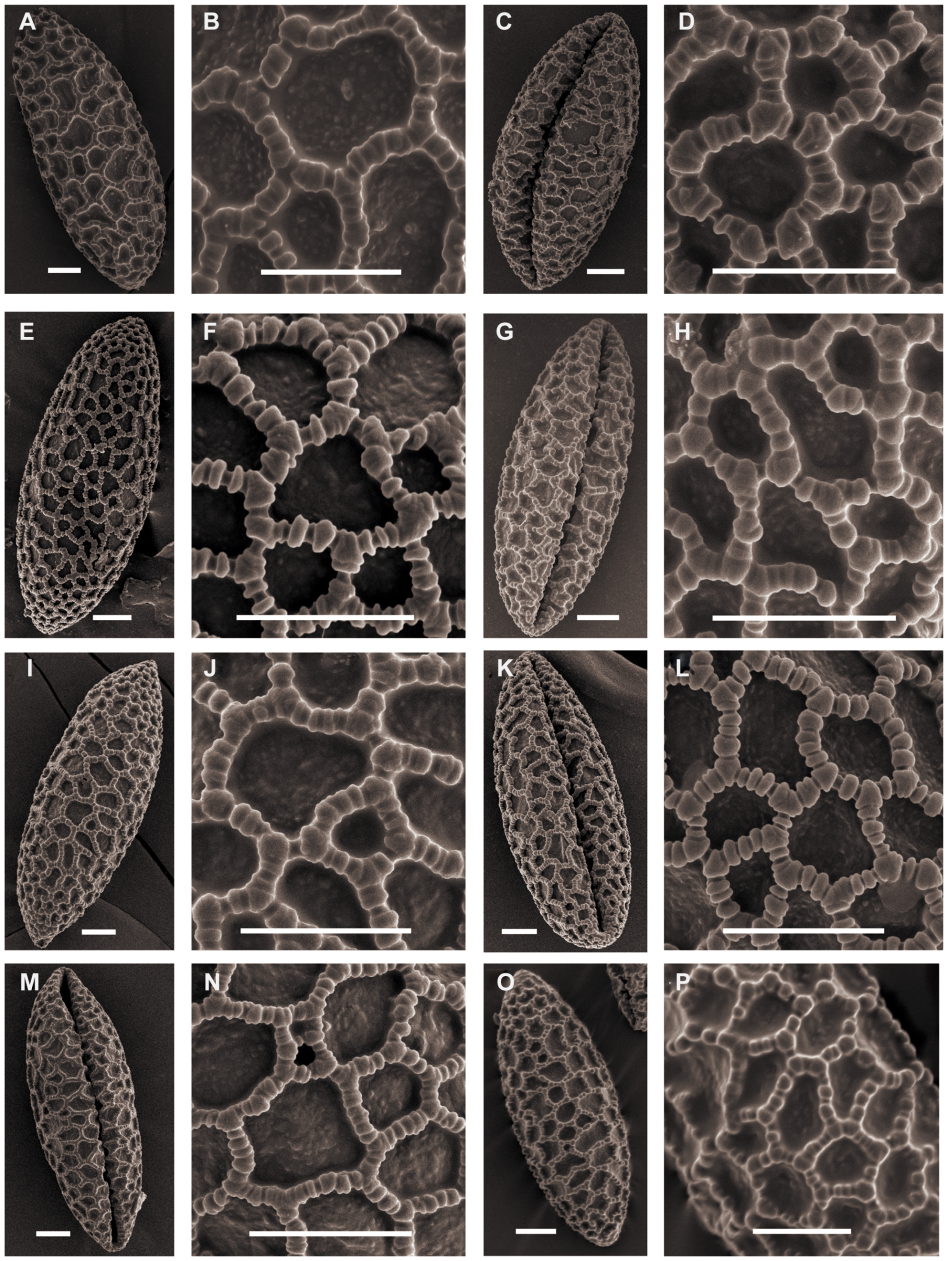
SEM photographs of pollen grains of *Lilium*. Figs A, B, *L. dauricum*. Figs C, D, *L. davidii* (Wang 11002). Figs E, F. *L. davidii* (Du 11008). Figs G, H, *L. davidii* (Wang 11003). Figs I, J, *L. davidii* (Du 11009). Figs K, L, *L. davidii* var. *willmottiae* (Jia 07002). Figs M, N, *L. davidii* var. *willmottiae* (Wang 11004). Figs O, P, *L. leichtlinii* var. *maximowiczii* (Du 12001). Scale bars: 10 µm.

Triploid *L. lancifolium* pollen is relatively larger than the other diploid species in section *Sinomartagon*. The shape can be ellipsoid to long-ellipsoidal. Species belonging to *Sinomartagon* (Figure 1M–P; 2A–P; 3A–P; 4A–P) show a decreasing tendency in size with regard to pollen features from subsect. 5a to subsect. 5c as follows: *L. lancifolium* Thunb (111.20×41.16 µm, P/E of 2.72); *L. davidii* Duchartre ex Elwes (93.09×33.55 µm, P/E of 2.79); *L. davidii* var. *willmottiae* (E. H. Wilson) Raffill (91.89×33.59, P/E of 2.75); *L. duchartrei* Franch (85.07×33.31, P/E of 2.56); *L. leichtlinii* Hook. f. var. *maximowiczii* (Regel) Baker (82.81×33.04, P/E of 2.52); *L. pumilum* DC (87.39×32.66, P/E of 2.67); *L. concolor* Salisb. var. *pulchellum* (Fisch.) Regel (70.77×30.58, P/E of 2.33); *L. wardii* Stapf ex Stearn (87.77×35.26, P/E of 2.49); and *L. taliense* Franch (74.70×29.70, P/E of 2.50) (Table 1).

**Figure 4:**
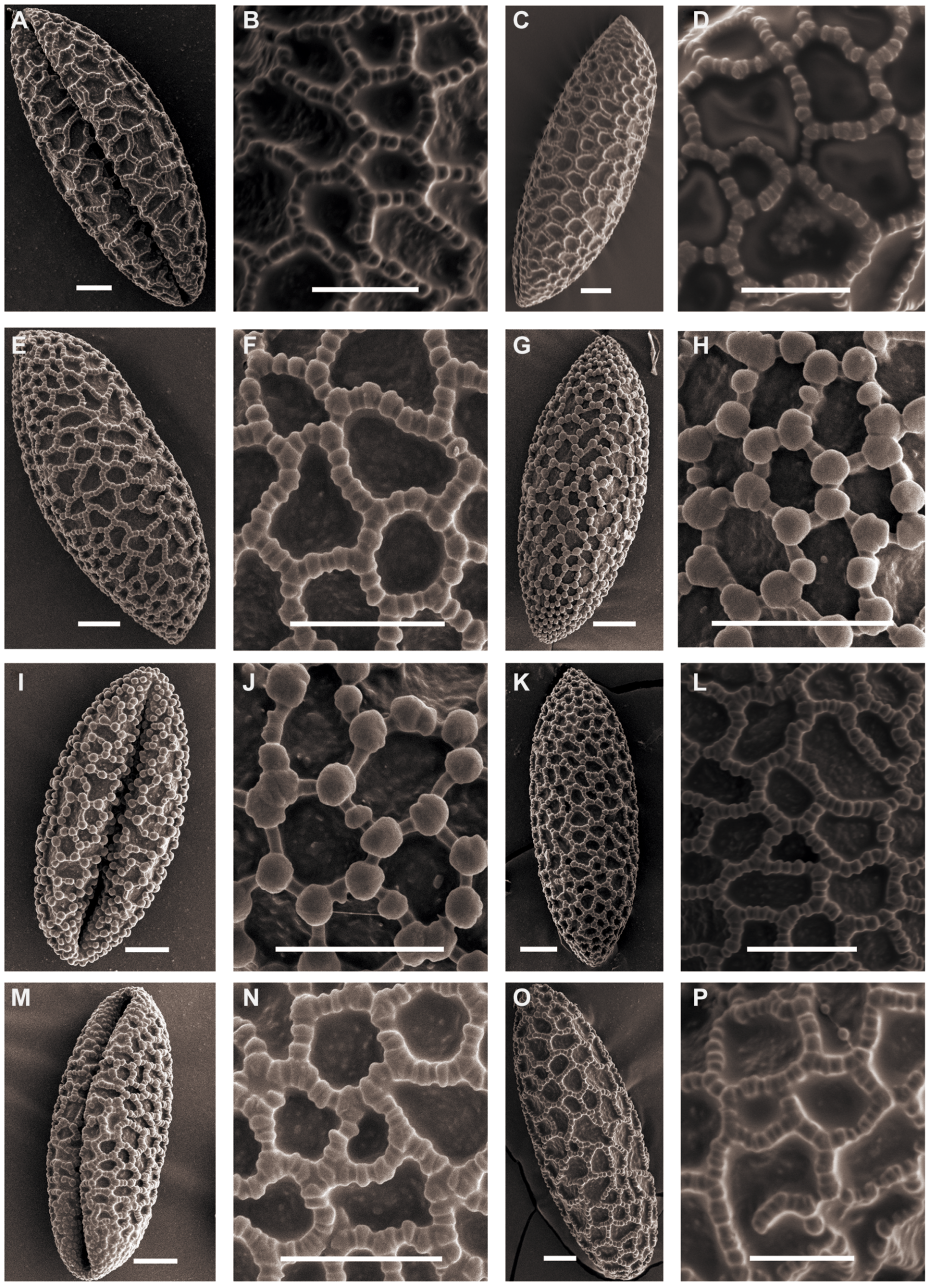
SEM photographs of pollen grains of *Lilium*. Figs A, B, *L. leichtlinii* var. *maximowiczii* (Du 11010). Figs C, D, *L.duchartrei*. Figs E, F, *L.pumilum*. Figs G, H, *L.concolor var. pulchellum* (Du 11011). Figs I, J, *L.concolor var. pulchellum* (Du 11012). Figs K, L, *L. taliense* (Jia 09002). Figs M, N, *L.taliense* (Du 12002). Figs O, P, *L.wardii*. Scale bars: 10 µm.

SEM of pollen from this section showed differences in exine sculpture. *L. concolor* var. *pulchellum* clearly shows Concolor type pollen (Figure 4E–H), whereas pollen from other *Sinomartagon* species show rectangular columellae that correspond to Martagon type.

### Leucolirion

**Figure 5:**
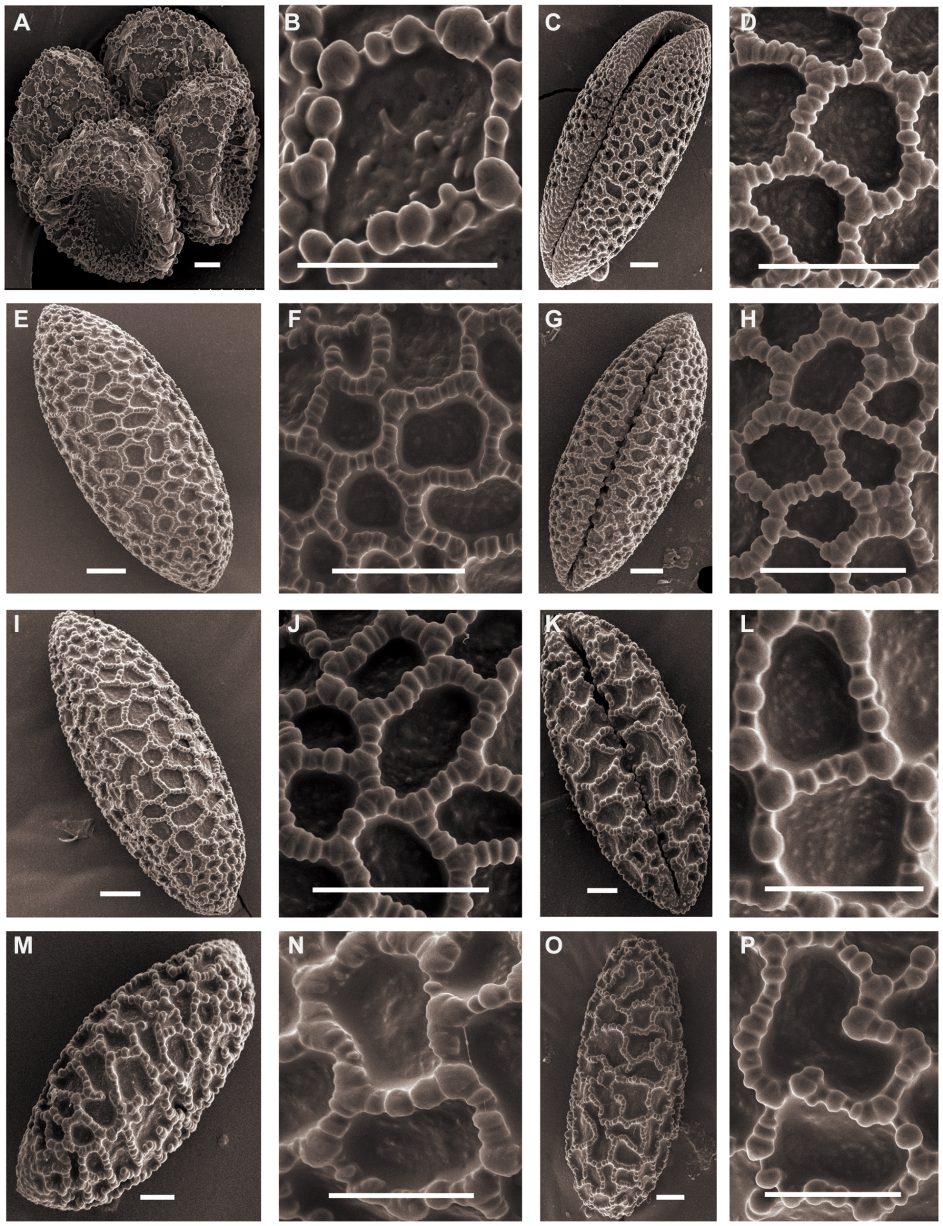
SEM photographs of pollen grains of *Lilium*. Figs A, B, *L.bakerianum*. Figs C, D, *L.henryi* (Du 11015). Figs E, F, *L.henryi* (Du 12004). Figs G, H, *L.rosthornii* (Jia 10001). Figs I, J, *L.rosthornii* (Du 12005). Figs K, L, *L. regale* (Du 11015). Figs M, N, *L. regale* (Wang 11007). Figs O, P, *L. regale* (Du 11016). Scale bars: 10 µm.

**Figure 6:**
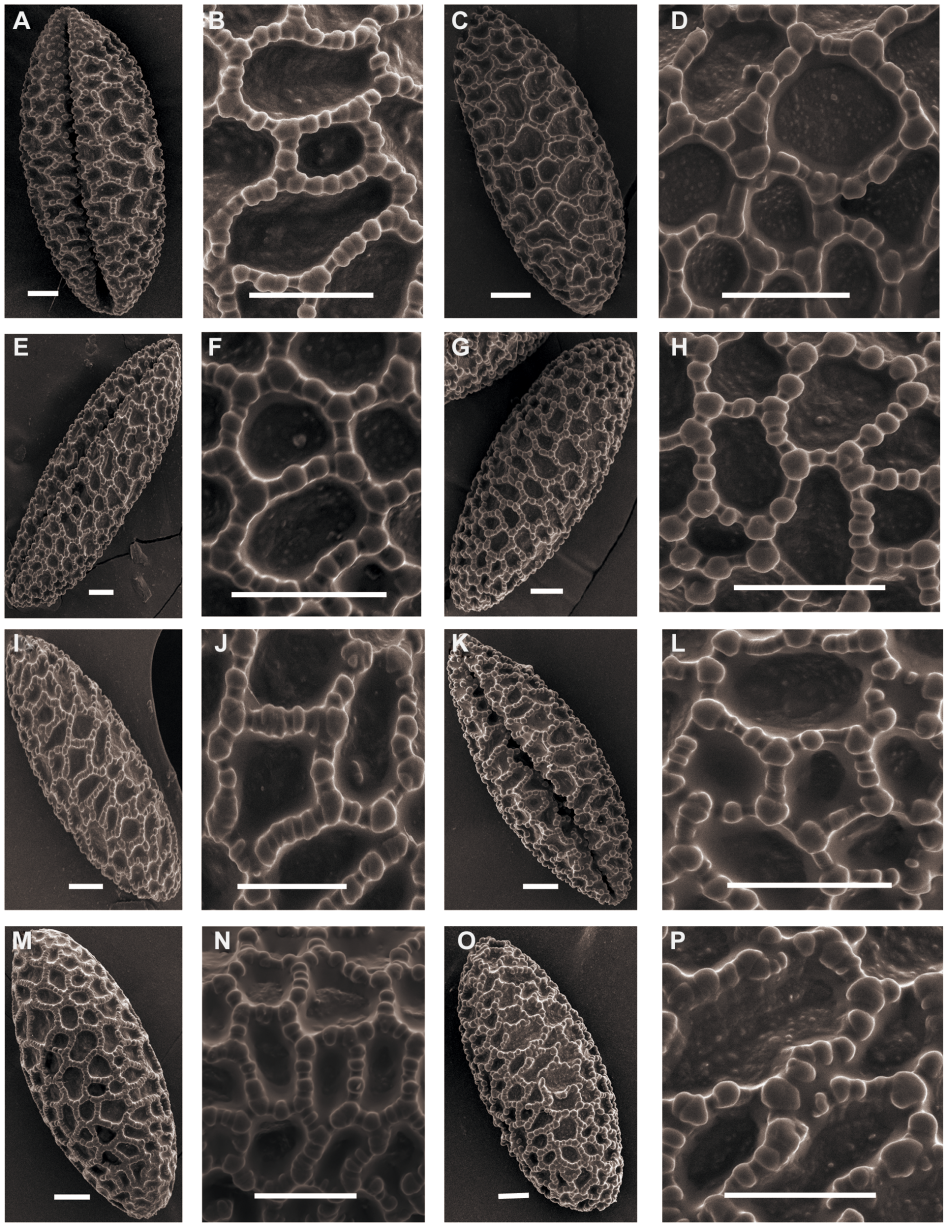
SEM photographs of pollen grains of *Lilium*. Figs A, B, *L. regale* (Du 12006). Figs C, D, *L.sargentiae* (Jia 11001). Figs E, F, *L.sargentiae* (Du 11017). Figs G, H, *L.sargentiae* (Du 11018). Figs I, J, *L.sargentiae* (Wang 11008). Figs K, L, *L. sargentiae* (Wang 11009). Figs M, N, *L. leucanthum*. Figs O, P. *L. sulphureum*. Scale bars: 10 µm.

**Figure 7:**
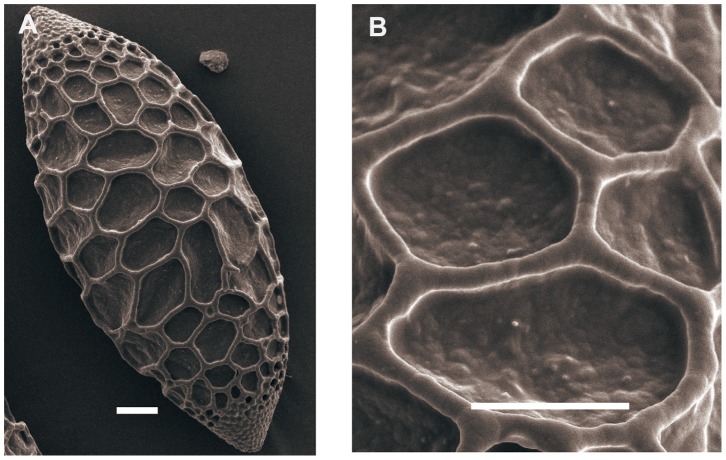
SEM photographs of pollen grains of *Lilium*. Figs A, B, *L. formosanum*. Scale bars: 10 µm.

Representatives of *Leucolirion* (Figure 1G–L; 5C–P; 6A–P; 7A–B) have pollen sizes ranging from 87.56×38.69, P/E of 2.28 for *L. sulphureum* Baker apud Hook. f. to 120.13×53.16, P/E of 2.29 for *L. brownii* from Shennongjia in the Hubei Province. The exine is ornamented with reticulation, and the shape is ellipsoidal to long-ellipsoidal. Pollen from *L. brownii*, *L. henryi* and *L. rosthornii* correspond to the Martagon type (Figure 1G–L; 5C–J). The pollen exine of *L. regale* Wilson, *L. leucanthum* (Baker) Baker, *L. sargentiae* Wilson and *L. sulphureum* show rounded columellae, corresponding to the Callose type (Figure 5M–P; 6A–P). Pollen of *L. formosanum* Wallace does not correspond to any of the pollen types proposed by Baranova (1985), and the exine surface is reticulate with solid muri or irregular-rugulate (Figure 7A–B). We suggest creating a new category of Formosanum type pollen.

### Lophophorum

The division of *Lophophorum* remains controversial. Due to resource constraints, we selected *L. bakerianum* as the only representative. In a preliminary study, *L. bakerianum* was placed in the subsect. *Lophophorum* III [16]. Pollen tetrads were found in *L. bakerianum* (Figure 5A–B), observed as blunt quadrangles in a polar view with a pollen size of 78.40×79.71 µm, P/E of 1.03. The exine ornamentation was reticulate with muri formed by separated rounded columellae, corresponding to the Callose type. This is the first study to report that pollen grains of *L. bakerianum* are tetrads.

## Discussion

### Pollen Morphology and Phylogenetic Implications Based on ITS Sequences

Within the genus *Lilium*, several diversified pollen exine sculptures were observed. Pollen sculpture patterns appear to reflect phylogenetic relationships and are useful for species or subsection delimitations within a section.

**Figure 8:**
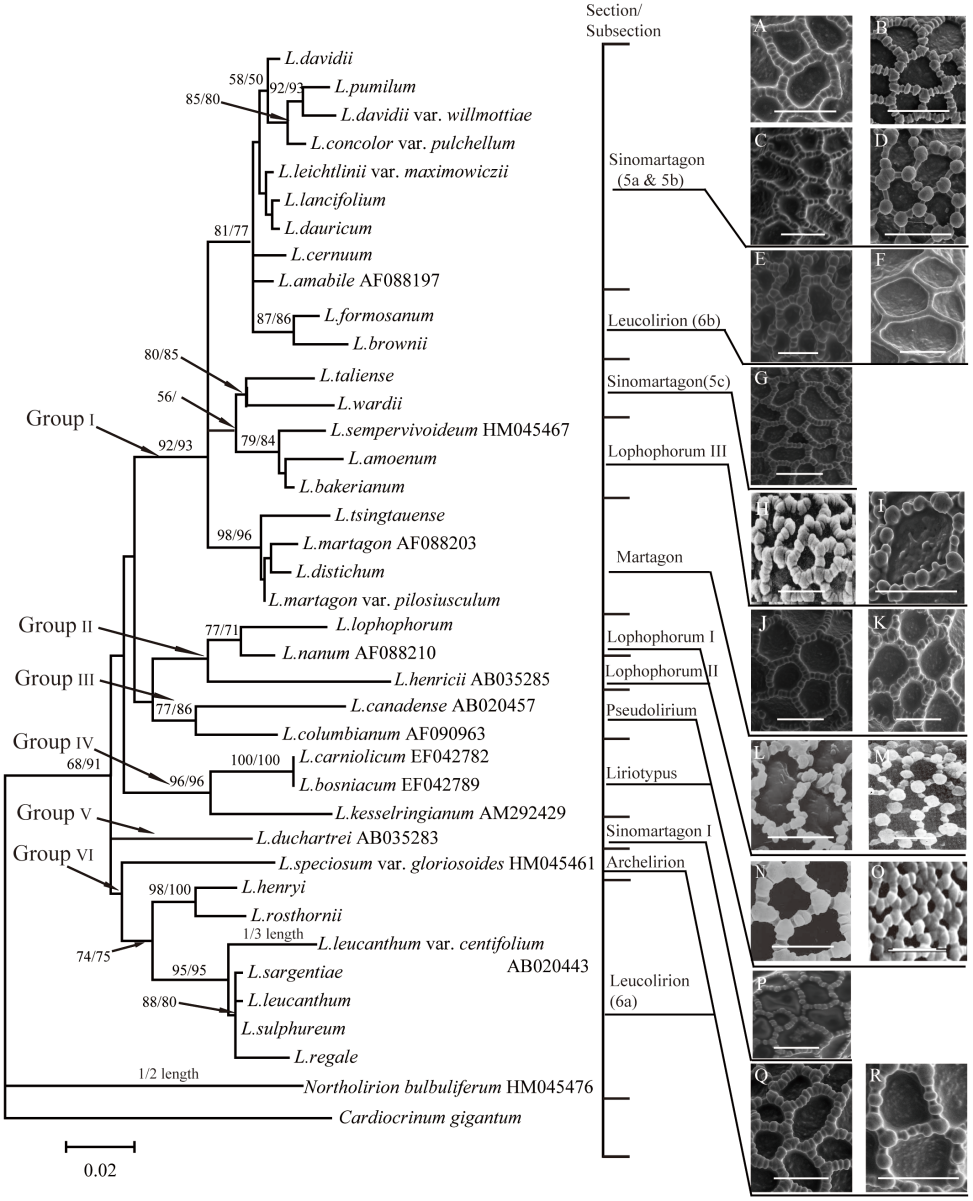
Mapping pollen exine sculpture characters on the ML tree of *Lilium* based on nuclear internal transcribed spacer (ITS) sequence data (adapted from Du et al. unpublished data). Values along branches represent bootstrap (BS) of ML and MP, respectively. A, B, C, D, Representatives of subsection 5a&5b. (A) *L.pumilum* (Jia 09001). (B) *L.davidii* (Du 11008). (C) *L. leichtlinii* var. maximowiczii (Du 11010). (D) *L. concolor* var. *pulchellum* (Du 11012). E, F, Representatives of subsection *Leucolirion* 6b. (E) *L.brownii* (Wang 11006). (F) *L.formosanum* (Du 12008). G, Representatives of subsection 5c (G) *L.taliense* (Jia 09002). H, I, Representatives of subsection *Lophophorum* III. (H) *L.sempervivoideum* (Liang and Zhang 1985). (I) *L.bakerianum* (Du 12003). J, K, Section Martagon. (J) *L.distichum* (Jia 07001). (K) *L.tsingtauense* (Du 11006). L, Representatives of subsection *Lophophorum* I. (L) *L. Lophophorum* (Liang and Zhang 1985). M, Representatives of subsection *Lophophorum* II. (M) *L. henricii* (Liang and Zhang 1985). N, Representatives of section *Pseudolirium*. (N) *L.canadense* (Kosenko, 1999). O, Representatives of section *Liriotypus*. (O) *L. bosniacum* (Muratović et al. 2010). P, Representatives of section *Sinomartagon* I. (P) *L.duchartrei* (Wang 11005). Q, Representatives of section *Archelirion*. (Q) *L. speciosum* var. *gloriosoides* (Wu et al. 2007). R, Representatives of subsection *Leucolirion* 6b. (R) *L. regale* (Du 11015).

The phylogenetic tree was resolved into six groups based on ITS sequences (Figure 8). Group I is comprised of three clades. Present and previous molecular analyses [18], [19] and cytology studies [21], [43]–[44] indicated that sect. *Sinomartagon* is complicated and polyphyletic, as supported by the pollen data (Figure 8). Species of sect. *Sinomartagon* (5a&5b) and *L. formosanum* and *L. brownii* (sect. *Leucolirion* 6b) formed a clade with strong support ([ML] BS = 81, [MP] BS = 77). Sect. *Sinomartagon* species show two pollen types, Martagon and Concolor. Differences in the shape and arrangement of the types of columellae could have implications on species delimitation. *L. concolor* var. *pulchellum* clearly shows Concolor type pollen (Figure 4G–J), and the remainder show Martagon type pollen. Meanwhile, three subtypes were showed within Martagon type pollen (Figure 8A, B, C). Exine sculptures of *L. davidii* and *L. davidii* var. *willmottiae* showed spherical but dorsally and ventrally compressed, and loosely arranged columellae with protuberant and enlarged columellae at the intersection of the muri (Figure 3B–N; 99B). Muri of *L. leichtlinii* var. *maximowiczii* have regular long-rectangular columellae (Figure 3O–P; 8C).

In addition, *L. formosanum* and *L. brownii* (*Leucolirion* 6b) formed a strongly supported clade ([ML] BS = 87, [MP] BS = 86) (Figure 8). In the cytological study, both *L. formosanum* and *L. brownii* lacked intercalary satellites on the first two pairs of chromosomes [43], [46] However, the pollen of *L. formosanum* corresponds to the new Formosanum type, while *L. brownii* clearly shows Martagon type pollen.

In our analysis, *L. taliense* and *L. wardii* ([ML] BS = 80, [MP] BS = 85) formed a clade with *L. amoenum*, *L. sempervivoideum* and *L. bakerianum* ([ML] BS = 79, [MP] BS = 84) that was moderately supported ([ML] BS = 56, [MP] BS<50) (Figure 8). In cytological studies, the *L. taliense* karyotype resembles that of *L. wardii* [21], [43]. Morphologically, *L. taliense* and *L. wardii* showed a close relationship with each other, with a black line in the central groove of the tepals [2]. Pollen evidence from our study supports this result, as the pollen morphology of the two species is generally similar Martagon type (Figure 4K–P). Morphologically, *L. amoenum*, *L. sempervivoideum* and *L. bakerianum* show nodding or horizontal campanulate flowers, nectaries of inner tepals neither papillose nor with fimbriate projections, tepals without blotch at the base adaxially, and a papillose stem [2]. The *L. bakerianum* var. *delavayi* karyotype resembles that of *L. sempervivoideum* [43]. Pollen tetrads are present in *L. amoenum*, *L. sempervivoideum* [23] and *L. bakerianum* (Figure 5A–B). The similarity in pollen morphology also suggests a close relationship with this clade.

The *Martagon* clade was monophyletic with strong support ([ML] BS = 98,[MP] BS = 96), which is consistent with previous studies by Gao et al. [13] and Nishikawa et al. [18], [19]. Morphologically, they all show whorled leaves. Although the *Martagon* clade shows Martagon type pollen, there are minor differences among species in the *Martagon* clade in the protuberant and enlarged columellae at the intersection of the muri in *L. tsingtauense* (Figure 1E–F, 8K).

Group II was weakly supported ([ML] BS<50, [MP] BS<50) and consisted of two *Lophophorum* clades. *Lophophorum* clade I consisted of *L. lophophorum* and *L. nanum* ([ML] BS = 77 and [MP] BS = 71). In palynological research, *L. lophophorum* and *L. nanum* are monocolpate or 2–3 porate and show similar pollen morphologies (Figure 8L) [24]. Representatives of *L. henricii* in subsect. *Lophophorum* II show Concolor type pollen with much smaller rectangular columellae between two separate irregular round columellae compared with *L. concolor* var. *pulchellum* (Figure 8M) [24]. Morphologically, representatives of the *Lophophorum* clade I show nectaries on the inner tepals with fimbriate/cristate projections on both surfaces, while representatives of subsect. *Lophophorum* II do not have this feature. The distinct pollen morphology of the species suggests a distinct relationship to support the molecular data [2].

In Group III, the *Pseudolirium* clade was resolved with strong support ([ML] BS = 77, [MP] BS = 86). Pollen morphologies from representatives of sect. *Pseudolirium* were similar, with loosely arranged columellae and protuberant and enlarged columellae at the intersection of the muri (Figure 8N) [35].

Group IV, consisting of *L. kesselringianum*, *L. bosniacum* and *L. carniolicum,* was robustly supported ([ML] BS = 97, [MP] BS = 97). The three representatives of European species (sect. *Liriotypus*) showed Martagon type pollen with densely arranged columellae forming the muri (Figure 8O) [25].

Group V was only comprised of *L. duchartrei* (*Sinomartagon* clade I) (Figure 8). Based on morphology, Comber classified it in subsection *Sinomartagon* 5a. However, in our ITS analyses, *L. duchartrei* showed a distant relationship with subsect. 5a species as well as other sect. *Sinomartagon* species (Figure 8), which is in accordance with previous studies [13], [18]–[19]. *L. duchartrei* (*Sinomartagon* clade I) karyotypes have been shown to be dissimilar to subsect. 5a *Sinomartagon* clade I species [21], [43]. *L. duchartrei* pollen resembles that of *L. taliense* and *L. wardii* in exine sculpture (Figure 4C–D; K–P). Given that the phylogenetic position of *L. duchartrei* is uncertain, classification as a subsection of sect. *Sinomartagon* may be reasonable.

In Group VI, *L. speciosum* var. *gloriosoides* in sect. *Archelirion* formed a weakly supported group consisting of representatives of *Leucolirion* 6a ([ML] BS<50 and [MP] BS<50). *Leucolirion* 6a comprised two subclades ([ML] BS = 74 and [MP] BS = 75). *L. speciosum* var. *gloriosoides* showed a distinct relationship with *L. henryi* and *L. rosthornii* in the ITS tree, although they all have Martagon-type pollen (Figure 8). In addition, *L. henryi* and *L. rosthornii*, which have orange reflexed flowers with prominent papillae and pubescent nectaries, were resolved with strong support ([ML] BS = 98, [MP] BS = 100). Species of *L. leucanthum*, *L. sargentiae*, *L. sulphureum* and *L. regale* formed a clade with strong support ([ML] BS = 95, [MP] BS = 95). Pollen morphology from our study supports this result, as the pollen morphology of *L. henryi* resembles *L. rosthornii* as Martagon type (Figure 5C–H), while *L. leucanthum*, *L. sargentiae*, *L. sulphureum* and *L. regale* have all generally similar Callose-type pollen (Figure 5K–L; 6A–P).

Within *Lilium*, we noted that one section may show two or more different pollen types (Figure 8). For instance, sect. *Sinomartagon* shows two exine sculpture pollen types, Martagon and Concolor, and the former contains three different subtypes. No unique and uniform characters can be used to clearly distinguish sections or some subsections due to shared or overlapping characters.

### Trends in Exine Sculpture Evolution and Factors Implicated in Pollen Evolutionary Trends

Changes in pollen morphology occur in response to selective pressure. Pollen grains should be considered a functional unit, with the exine structure as a compromise between the following four main functions [32]: protective, harmomegathic, reservoir and clustering. These functions are in response to physical components of the environment, such as water, nutrients, temperatures and growing season.

The genus *Lilium* is distributed in East Asia, the Himalayas and Hengduan Mountains, Europe and North America [1], [2]. Patterson and Givnish [4] concluded that *Lilium* evolved in the Himalayas and then dispersed into Eurasia and North America, although intercontinental dispersal details are not clear [4]. In the karyotype study, the relatively primitive group (sect. *Lophophorum* (Bur. et Franch.) Wang et Tang) have no intercalary satellites on the first two pairs of chromosomes. As expected, the genus *Notholirion* which is commonly considered to be closely related to and more ancient than *Lilium* have also been found to lack intercalary satellites [4], [21], [43], [45], [46], these species are distributed mainly in the Himalayas, which makes this region more likely as the origin of the genus *Lilium*. Besides, Stewart [45] pointed out that secondary constrictions correlated with chromatin distribution as well as with geographic distribution. In his study, most East Asian and Himalayan species were found to have intercalary satellites on the first two pairs of submedian centromeres, while all North American species and European species lack such feature [21], [25], [43], [45,]. Hence, the situation among the Himalayan and Henduan Mountains species appears more complicated since they seem to have both types, even within the same section indicating that the Hengduan Mountains are more likely as the differentiation centre of the genus [43], [45], [47]. Pollen evidence from our study supports this hypothesis, as the pollen morphology of the species from the Himalayas and Hengduan Mountains contain all of the pollen types in the genus *Lilium*. It should be emphasized that the Formosanum type of pollen can be found in the genus *Nomocharis,* which is restricted to the Himalayas and Hengduan Mountains and it has been suggested that it be included in *Lilium* [13]–[16]. In contrast, the other regions possess a relatively singular pollen type, which led us to investigate *Lilium* pollen-type evolution.

Based on a pollen study of 30 genera in Liliaceae, including 69 species, using light microscopy (LM), Nair and Sharma [46] proposed the scheme of pollen exine sculpture evolution (Liliaceae) as follows: ornate → retipilate → reticulate → ring-shaped reticulate → scattered reticulate → exineless ornamentation. Regarding pollen morphology of the genus *Nomocharis* and *Lilium* under LM and SEM, Liang and Zhang [24] suggested the following evolution trend of pollen exine sculpture in the genus *Lilium*: ornate → retipilate → reticulate, which is consistent with the hypothesis of Nair and Sharma [46]. In addition, retipilate was divided into three exine sculptures to form three *Lilium* pollen types, including Martagon, Callose and Concolor. These results led us to propose the following hypothesis of pollen evolution in exine sculptures within *Lilium*: Martagon type → Callose type → Concolor type → Formosanum type. This also shows a reduction and simplification tendency.

In monocots, reduction and simplification of the exine structure is even more extreme than in dicots and culminate in certain evolutionary orders [48]. Zavada proposed major evolutionary trends of wall structure types in monocots as follows: primitive tectate-columellate (perforate or imperforate) wall structures give rise to monocotyledonous atectate or granular walls and eventual extreme reduction of the exine, which may be completely absent [48]. All morphological characteristics are the consequence of interactions between phylogenetic and environmental constraints [32]. There is no doubt that environmental constraints act on patterns of differentiation in *Lilium* [49].

In addition to environmental factors, are there other factors such as pollinator affecting pollen differentiation? Numerous recent findings on the intricate flower pollinator networks suggest that differentiation of flora structures and functions in plants are tightly connected with those of pollinators [50]–[53]. Flowers of *Lilium* are insect-pollinated. Brantjes and Bos’s [54] report that both diurnal and nocturnal hawkmoths visit *L. martogon*. Skinner [55] observed pollinators of 13 American species of *Lilium* and recognized five types of pollination: butterfly pollination (*L. humboldtii*, *L. kelleyanum* and *L. kelloggii*), butterfly and hummingbird pollination (*L. wigginsii*, *L. pardalinum* and *L. pardalinum* ssp. *vollmeri*), hummingbird pollination (*L. columbianum*, *L. occidentale* and *L. bolanderi*), hummingbird and bumblebee pollination (*L. parvum* and *L. maritimum*) and hawkmoth pollination (*L. washingtonianum* and *L. parryi*). Additional studies reported various flower visitors, including fritillaries to *L. concolor* var. *pulchellum* [56], swallowtails to *L. dauricum* [56], nocturnal hawkmoths to *L. formosanum* [57] and *L. japonicum* [58], and hawkmoths and swallowtails to *L. auratum* [56], [59]. Nevertheless, wild *Lilium* species are self-compatible. Autogamy might have ensured reproductive success for the species in the environments where pollinators are comparatively rare [60] and the growing season short as well as where other conditions are selective. Pollen of *L. martagon* and *L. dauricum* with different pollinators are Martagon type (Table 1) [54], [56]. Thus, for *Lilium*, the pollinators have little effect on changes of pollen morphology and size. Moreover, in Sonneratiaceae, the genus *Duabanga* has much smaller pollen grains than *Sonneratia* while both genera are bat-pollinated, hence absence of correlation with pollinator [32]. However, how floral traits as well as pollen grains of lilies are adapted to various pollinators remains to be studied in more detail.

As mentioned, environmental factors constitute a selective pressure that may produce changes in the pollen structure. The number and arrangement of the types of columellae could influence exine flexibility. Exines with loose arrangement of round columellae (Callose, Concolor) are relatively more flexible compared to exines with tight, rectangular columellae (Martagon). This exine structure corresponds to harmomegathic function and the ability to absorb bending stresses that may occur during desiccation [32].

### Relationship between Pollen Size and Conditions in the Genus *Lilium*

Species at high elevations with relatively extreme conditions in the Himalayas and Hengduan Mountains have a relatively small pollen size (Table 1). Plants growing at high elevations with extreme conditions often have reduced morphological features [61], [62], and pollen size also appears to show this tendency [33], [63]. Furthermore, species native to Northeast China, such as *L. leichtlinii* var. *maximowiczii*, *L. concolor* var. *pulchellum* and *L. dauricum,* also have a relatively small pollen size (Table 1). A long winter and short growing season forces rapid completion of plant growth cycles. Hence, small pollen size also appears to be more efficient in tolerating relatively extreme conditions. In contrast, *L. brownii* and *L. formosanum* have relatively bigger pollen grains, likely because they occur in an environment suitable for slow development that allow pollen grains to grow to a larger size, as discussed by Muller [32]. Therefore, pollen size could be related to the selective pressure to adapt to environmental conditions. In addition, smaller pollen grains appear to survive better in habitats with extreme conditions.

Within the same species, pollen parameters from different provenances showed some variation (Table 1). Based on one-way ANOVA and LSD tests in 11 species (Table 2), results showed significant or extremely significant differences in pollen parameters in the same species from different habitats within the provenances, except for several single parameters in some species (Table 2). This may be explained as a consequence of interactions between phylogenetic and environmental constraints, as emphasized by Kawano and Kato [31].

Due to the wide variety of habitats in the distribution of *Lilium*, correlation analysis was performed to test which environmental factor is critical in influencing pollen parameters. The correlation analysis showed a significantly positive correlation between the polar axis and annual precipitation (P<0.05) and an extremely significantly positive correlation between the P/E ratio and annual precipitation (P<0.01) (Table 4). Hence, pollen size and shape showed a significantly positive correlation with annual precipitation. The absolute size of pollen grains influences harmomegathic function [64], which can be reduced to such a degree that exine elasticity alone can accommodate the necessary changes in volume to adapt to environmental conditions [30].

Pollen exine sculptures of different species in similar environmental conditions may be different types. For instance, *L. concolor* var. *pulchellum* possesses Concolor type pollen, whereas *L. dauricum* shows Martagon type pollen, and both occur in Northeast China under similar environmental conditions. Species native to the Himalayas and Hengduan Mountains show all of the pollen types within *Lilium*. The evolutionary trend of exine sculptures is likely not definitively correlated with pollen size.

## Conclusion

In conclusion, we suggest recognising a new pollen type, Formosanum, to accommodate pollen from *L. formosanum*. Pollen sculpture patterns appear to reflect phylogenetic relationships and are useful for species or subsection delimitation within sections. The reduction and simplification evolutionary trend of pollen sculpture and size could be related to the selective pressure to better adapt to environment conditions, especially extreme environmental conditions. In addition, the evolutionary trends of exine sculpture and pollen size and shape are not definitively correlated. Pollen size and shape show a significantly positive correlation with annual precipitation. However, additional studies are required to confirm these evolution hypotheses in a broad sample of the genus using techniques such as transmission electron microscopy (TEM) to observe strata of the exine.
