# Correction: *Lilium* spp. pollen in China (Liliaceae): Taxonomic and Phylogenetic Implications and Pollen Evolution Related to Environmental Conditions

**DOI:** 10.1371/journal.pone.0091543

**Published:** 2014-03-07

**Authors:** The PLOS ONE Staff

Affiliation number 1 for all authors is incorrect.

The correct affiliation is:

Beijing Key Laboratory of Ornamental Plants Germplasm Innovation & Molecular Breeding, National Engineering Research Center for Floriculture and College of Landscape Architecture, Beijing Forestry University, Beijing, China.
